# Matching the Rheological Properties of Videofluoroscopic Contrast Agents and Thickened Liquid Prescriptions

**DOI:** 10.1007/s00455-012-9441-x

**Published:** 2013-02-14

**Authors:** S. Popa Nita, M. Murith, H. Chisholm, J. Engmann

**Affiliations:** Nestlé Research Center, Nestec Ltd, Vers-chez-les-Blanc, PO Box 44, 1000 Lausanne 26, Switzerland

**Keywords:** Videofluoroscopic contrast agents, Deglutition, Deglutition disorders, Thickener, Viscosity, Shear thinning

## Abstract

In the treatment of oropharyngeal dysphagia, the link between diagnosis and prescription of thickened liquids that are safe to swallow is not always straightforward. Frequently, the capacity to objectively assess and quantify the rheological properties of diagnostic test fluids and to select “rheologically equivalent” dietary products is missing. Perhaps sometimes the importance of an objective comparison is not fully appreciated because two liquids seem reasonably similar in a subjective comparison (e.g., flow from a spoon). The present study deals with some of these issues. Shear viscosity measurements were used to characterize the flow behavior of videofluoroscopic contrast agents and of thickened fluids prepared with commercial thickening agents. Effects of time and composition of the different fluids were analyzed regarding shear-rate-dependent viscosity. Nearly all materials tested showed a pronounced dependence of viscosity with shear rate (“shear thinning”). Results confirm that it is feasible (but not always straightforward) to “match” the viscosities of diagnostic fluids and thickened beverages if certain precautions are taken. For example, the time required to reach final viscosity levels can be significant for some thickeners, particularly when used with liquids containing contrast agents. It is recommend to use only diagnostic materials and thickening agents for which reliable viscosity data are available.

## Introduction

For many individuals suffering from dysphagia, their capacity to swallow safely and effectively is compromised for a certain range of bolus consistencies, e.g., thin liquids below a certain viscosity. It is difficult to determine a safe range of consistencies quantitatively, even if a careful diagnostic procedure (using materials with different viscosities) is conducted to identify this safe range and to recommend adequate dietary restrictions [1, 2].

Challenges originate partially in the fact that every person has slightly different abilities to differentiate bolus consistencies and uses different expressions to describe them. What is a “thin liquid” for one person may not appear “thin” to someone else, unless uniform standards and terminologies are used. As a consequence, it can be a serious problem to recommend a specific range of consistencies for a patient suffering from dysphagia, particularly if the person performing the diagnostic test (e.g., using videofluoroscopy) is not the person selecting and prescribing the diet. Recent studies [3–6] have strikingly shown the lack of agreement between different (trained) individuals when judging the consistency of diagnostic test fluids and/or products. Even if the same person is making the comparison (e.g., by stirring, pouring, or tasting a liquid or actually making the product), the result is not always reproducible [4].

The characterization and prediction of flow properties such as shear viscosity are known as the science of rheology [7–9]. By using appropriate measurement techniques, one can unambiguously determine the viscosity of different materials and thus avoid “comparing apples with pears” [10, 11].

In a first step toward a more objective comparison, several national groups (e.g., the National Dysphagia Diet (NDD) Task Force in the US, the British Dietetic Association, or the Dietitians and Speech Pathology Associations of Australia) have developed standards to classify bolus consistencies [12–15]. In doing so, it has been appreciated that for products more viscous than water (referred to, e.g., as “nectar-like,” “honey-like,” and “spoon-thick”), the viscosity depends not only on temperature (as for all liquids), but also on the speed of flow, or shear rate. Thickened products are typically seen to shear thin, i.e., the force required to keep them flowing grows less than proportional with the shear rate. The viscosity, defined as the ratio of shear stress (the force required for flow) and shear rate (related to the flow rate) therefore decreases with increasing shear rate.^1^

As a consequence, the NDD standard [14] specifies a shear rate at which viscosity needs to be evaluated to allow a meaningful comparison. Instruments employed to determine the shear viscosity must be able to accurately establish this shear rate (which may not always be realized in practice).

It is not fully understood which range of shear rates constitutes the most representative conditions with respect to mastication and swallowing processes [16], but the current understanding suggests that the NDD standard of 50 s^−1^ (50 per second or 50 reciprocal seconds, equivalent to a change in velocity from 0 to 50 mm/s over a distance of 1 mm) is at least a reasonable order of magnitude with respect to in-mouth handling of the bolus. The limitations of this shear rate value are well recognized and changes are expected to occur as more research is performed in the future [14].

In this work, shear viscosity measurements were performed over a range of shear rates, typically from 0.01 to 100 s^−1^, i.e., covering four orders of magnitude, for a variety of commercially available diagnostic test materials used in videofluoroscopy (modified barium or iodine swallow). These materials contain contrast agents such as barium sulfate particles (BaSO_4_) or soluble iodine complexes (Natrii amidotrizoas, Meglumini amidotrizoas), and although these substances affect viscosity only weakly (at least at low to moderate concentration levels), they can interact with other materials added to modify viscosity, such as starch or gums [17].

Since diagnostic test materials are sometimes thickened by the addition of food thickeners like starch or gum to widen the range of consistencies during an examination, measurements of shear viscosity for such mixtures were also performed and attention was given to practical aspects such as thickening time [11, 16, 18–20]. If these points are not taken into consideration, the true shear viscosity of the materials being compared could be strongly over- or underestimated.

Additional rheological and physical properties may affect swallowing performance. *Density* is certainly altered by the addition of contrast materials, and previous studies showed that increased density can lead to difficulty in swallowing [21]. However, to our knowledge, no systematic work has been carried out so far to study the effect of density independent of rheological effects [i.e., using fluids with the same rheological properties (viscosity, yield stress) and different densities]. It should also be noted that the density of foods, beverages, and diagnostic materials varies within a relatively narrow range of approximately 1–2 g/ml, whereas viscosities easily range from 1 mPa s (1 cP) to many thousands of mPa s, even for easily flowable liquids. The *yield stress* of a bolus (the level of force required to initiate flow) may also play an important role, but this property is much harder to measure unambiguously than viscosity, as it is intrinsically linked to the flow history of the material. Different protocols can therefore provide quite different results [22]. However, more attention should be given to this property and its effects on swallowing in the future.

## Materials

The videofluoroscopic contrast agents studied and their main characteristics are listed in Table 1. Barium sulfate-based contrast agents (E-Z-Paque^®^ and the Varibar^®^ series) were purchased from E-Z-EM, USA, and the iodine-based Gastrografin^®^ was purchased from Bayer, Switzerland. These two types of contrast agents were chosen as they have already been used in several published studies on oropharyngeal swallowing disorders [21, 23].

**Table 1:** Videofluoroscopic contrast agents used in the present study

Name	Concentration of contrast substance (%w/w)	Target viscosity (mPa s)	Viscosifying agents (thickeners)
Varibar^®^ Thin Liquid	–	4	Carboxymethyl cellulose
Varibar^®^ Nectar	30	300	Carboxymethyl cellulose, natural gum
Varibar^®^ Thin Honey	29	1,500	Carboxymethyl cellulose, natural gum, modified starch (corn)
Varibar^®^ Honey	29	3,000	Carboxymethyl cellulose, natural gum, modified starch (corn)
Varibar^®^ Pudding	30	5,000	Carboxymethyl cellulose, natural gum
E-Z-Paque^®^	41	–	–
Gastrografin^®^	26	18.5	–

The information in columns 2–4 is given by the suppliers. Viscosity values are given in mPa s (millipascal seconds) and are equivalent to cP (centipoise). According to the supplier, for the Varibar^®^ series of products, viscosity values are valid at 30 s^−1^ and 25 °C

Two commercially available thickeners designed specifically for patients with dysphagia were used in this study: a conventional starch-based thickener TU (Resource^®^ ThickenUp™, Nestlé HealthCare Nutrition, Nestec S.A., Switzerland) and a xanthan gum-based thickener TUC (Resource^®^ ThickenUp™ Clear, Nestlé HealthCare Nutrition). Manufacturer recommended dosages for the two thickeners are shown in Table 2. Where needed, mineral water Vittel Bonne Source (Nestlé, France) was used for sample preparation.

**Table 2:** Recommended dosages for 100 ml of water using TU and TUC thickening agents

Required consistency	Nectar-like (g)	Honey-like (g)	Pudding-like (g)
Resource^®^ Thicken Up™ (TU)	4.5	6.75	9
Resource^®^ Thicken Up™ Clear (TUC)	1.2	2.4	3.6

## Methods

**Fig. 1:**
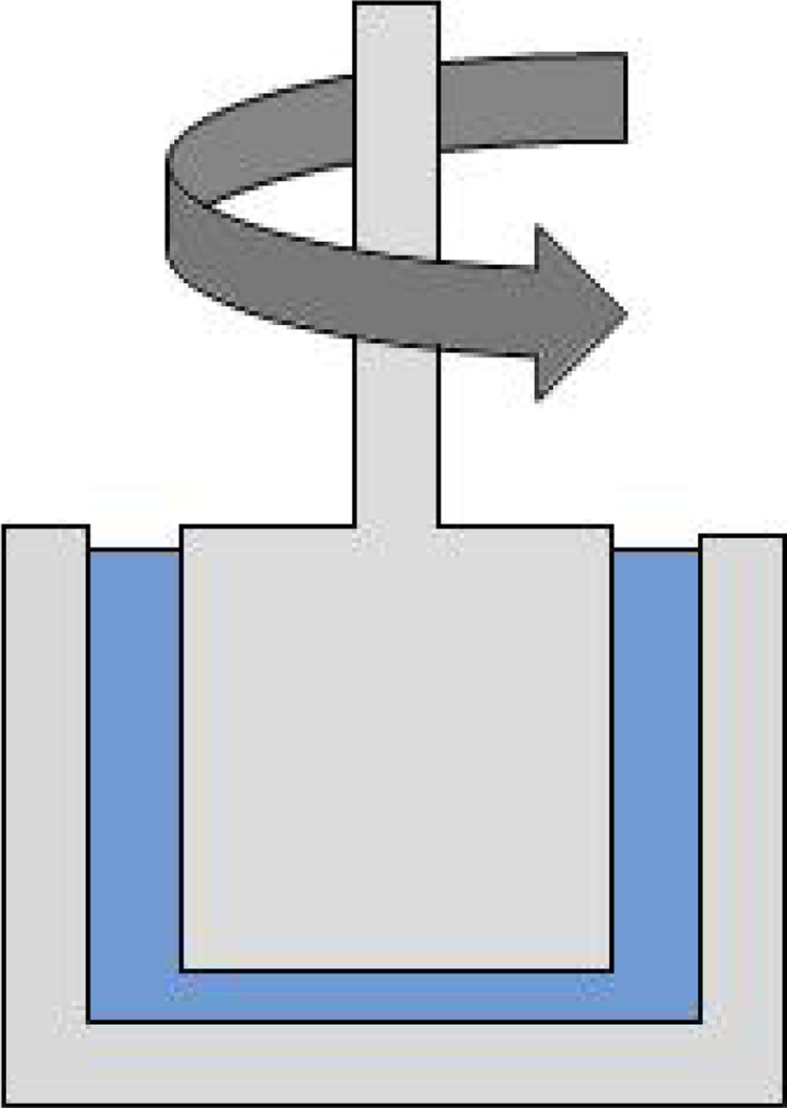
Schematic view of the concentric cylinder geometry used in this study

Viscosity variation with shear rate was measured using a MCR 500 rheometer (Anton Paar) equipped with a concentric cylinder geometry CC27, with a measuring gap of 1.13 mm and a gap length of 40 mm. The advantage of this geometry is that the shear rate is effectively constant throughout the sample volume and that low-viscosity fluids can be measured (thanks to a large fluid/geometry contact area relative to the sample volume). A schematic view of the concentric cylinder or “cup and bob” geometry is shown in Fig. 1. The fluid to be tested is confined in the narrow gap between the two cylinders. The torque required to turn the bob at a constant angular velocity is measured and related to shear stress and hence viscosity. The shear rate interval investigated was 0.01–100 s^−1^ to cover a wide range of shear rates thought to be relevant, as experienced by the bolus during the swallowing process (from the oral cavity to passage through the oropharynx). Throughout each measurement, the temperature of the sample was maintained at 20 °C by a Peltier module.^2^ In general, at least three independent measurements were performed and the calculated average is presented.

Thickener dispersion in water or videofluoroscopy contrast fluids was achieved by following supplier instructions (e.g., stirring with a spoon or shaking). No powder clumping was observed as long as a vortex was created in the liquid with a spoon prior to addition of thickener powder and the powder was added slowly under continuous mixing.

## Results

### Contrast Materials and Thickener Solutions of Different Consistencies

**Fig. 2:**
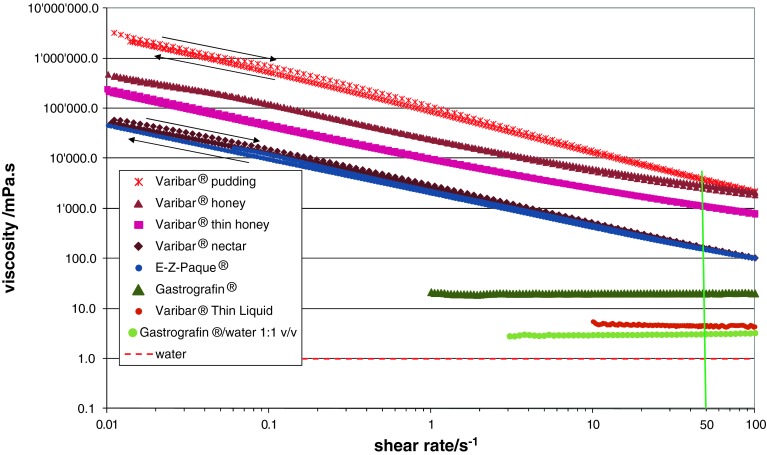
Shear viscosity variation with shear rate for different videofluoroscopic contrast agents

The number of diagnostic test materials for videofluoroscopy (modified barium or iodine swallow) available on the market is large and the proposed consistencies can be very different. Figure 2 shows the variation of the shear viscosity as a function of shear rate for seven liquid contrast materials, all measured at a temperature of 20 °C (where the viscosity of pure water is 1 mPa s), first at increasing shear rates (from 0.01 to 100 s^−1^) and then decreasing ones (from 100 to 0.01 s^−1^). The shear rate of 50 s^−1^ (specified by the NDD guidelines) is highlighted, as this is where materials should be compared according to the NDD standard.

**Fig. 3:**
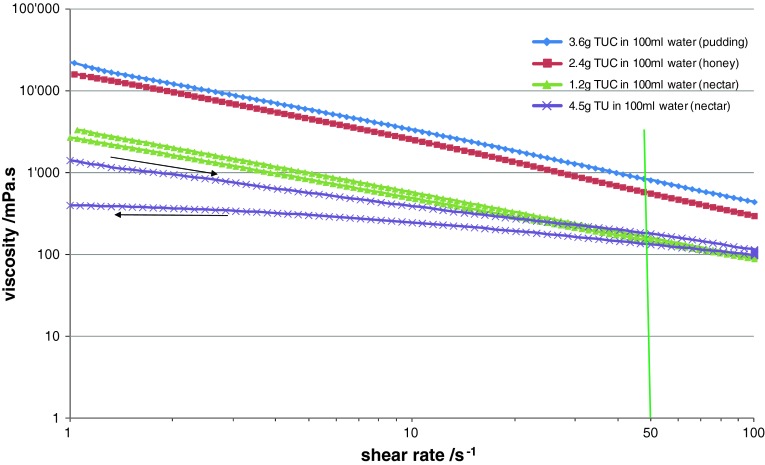
Viscosities of thickened solutions at different consistencies (nectar, honey, pudding) obtained using two commercial thickeners: Resource^®^ ThickenUp™ Clear (TUC) and Resource^®^ ThickenUp™ (TU). Solution concentrations were chosen following recommended dosages of the thickener products (see Table 2)

Data for two commercial thickeners (starch-based TU and xanthan gum-based TUC) used to modify beverage and food consistencies for people suffering from dysphagia are shown in Fig. 3. For TUC, data are shown at several different concentrations corresponding to the different levels: nectar-like, honey-like, and pudding-like (see Table 2).

### Matching Viscosities of Diagnostic Fluids and Thickener Solutions

**Fig. 4:**
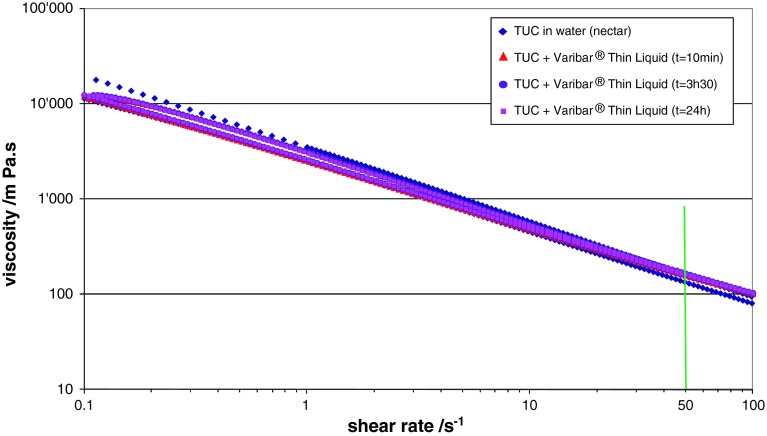
Viscosities of solutions of Resource^®^ ThickenUp™ Clear (TUC) reconstituted in water and in Varibar^®^ Thin Liquid at nectar consistency (i.e., 1.2 g of TUC for 100 ml liquid)

**Fig. 5:**
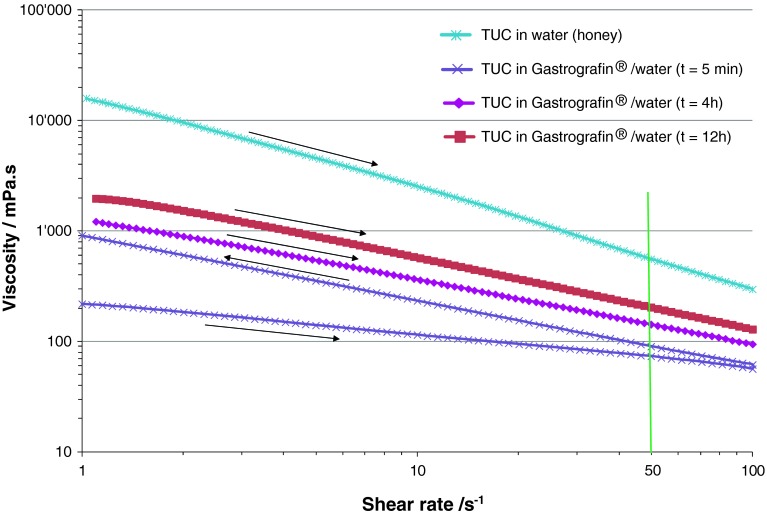
Viscosities of solutions of Resource^®^ ThickenUp™ Clear (TUC) reconstituted in water and in Gastrografin^®^/water (1:1 volume ratio) at honey consistency (i.e., 2.4 g of TUC for 100 ml liquid)

**Fig. 6:**
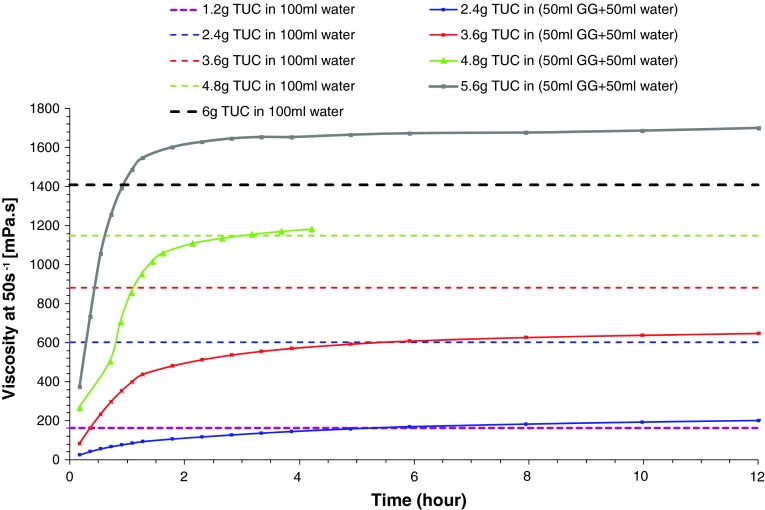
Build-up of viscosity of solutions of Resource^®^ ThickenUp™ Clear reconstituted in Gastrografin^®^/water. Viscosity values measured at a shear rate of 50 s^−1^ are plotted against time. *Dashed lines* represent the viscosities at a shear rate of 50 s^−1^ of TUC solutions prepared with water only. *GG* Gastrografin^®^

The thickening of contrast agents to different consistencies by using food thickeners was studied in the case of Varibar^®^ Thin Liquid and Gastrografin^®^ mixed with a xanthan-based thickener, TUC. Very different behaviors were observed: in the case of Varibar^®^ Thin Liquid, a straightforward match of viscosity was obtained between thickened contrast agent and thickened water and the viscosity values were constant over time (Fig. 4). On the other hand, in the case of Gastrografin^®^/TUC mixtures, measured viscosities varied greatly over time and matching them with those of water/TUC mixtures required an extensive rheological study (Figs. 5, 6).

## Discussion

### Contrast Materials and Thickener Solutions of Different Consistencies

The viscosity variation with shear rate, over a wide range of shear rates thought to be relevant as experienced by the bolus during the swallowing process, was studied for a large number of videofluoroscopic contrast fluids available on the market (Fig. 2). It is readily seen that comparing viscosities not obtained at the same shear rate can lead to highly misleading conclusions, e.g., even the Varibar^®^ pudding consistency has a lower shear viscosity measured at a high shear rate than the Varibar^®^ nectar consistency measured at a low shear rate! In general, a strong decrease in viscosity with increasing shear rate (i.e., shear-thinning) is observed for all the materials except for Varibar^®^ Thin Liquid and Gastrografin^®^ (pure or diluted with water).

When comparing viscosity values at 50 s^−1^, all grades of the Varibar^®^ range are clearly distinct and the order corresponds to the different stated levels; however, some of the denominations (e.g., Varibar^®^ honey) do not correspond to the range defined by the NDD standard (Table 3). This observation is also valid when viscosity values at 30 s^−1^ are considered (as specified by the manufacturer). Due to different shear-thinning behaviors, fluids like Varibar^®^ pudding and Varibar^®^ honey have significantly different viscosities at low shear rates, but at shear rates above 50 s^−1^, the differences are within 25 % variation and lower. Moreover, E-Z-Paque^®^ has a viscosity essentially identical to the Varibar^®^ nectar and thus is substantially more viscous than water. It should therefore not be considered a “thin liquid” in swallowing studies, as its viscosity is at least 100 times higher than water for shear rates up to 100 s^−1^. The fact that all materials measured showed—within the precision of measurement—no differences between shear viscosity at increasing and decreasing shear rate means that they do not exhibit shear history dependence: the shear viscosity is influenced by the shear rate (speed of flow), but not by how this shear rate is reached, an important point for consistent performance of these fluids during examination. For example, if a test sample is administered to a patient using a syringe (to control its volume), this sample is subjected to very high shear rates in the tip of the syringe. If the sample has a shear history dependence, then its behavior during swallowing and the viscosity perceived by the patient might be different from the case where the sample was administered from a spoon. Minimizing effects of shear history dependence is thus very important.

**Table 3:** Standard classification of bolus consistencies defined by the National Dysphagia Diet Task Force (NDD standard)

Consistency	Thin	Nectar	Honey	Spoon-thick
Viscosity range (mPa s)	1–50	51–350	351–1,750	>1,750

Viscosities need to be evaluated at a shear rate of 50 s^−1^ and temperature of 25 °C to comply with these standards

For commercial thickeners dispersed in water (Fig. 3), the dependence of viscosity on shear rate is similarly strong as for the more viscous contrast materials. As in the case of the xanthan gum-based thickener (TUC), the viscosity increases with increasing thickener concentration in solution (here from 12 to 36 g/l). TUC has a higher thickening effect than the starch-based thickener (TU) as comparable viscosities are obtained for solutions of 12 g/l of TUC and 45 g/l of TU. The two thickeners are also different in their shear history dependence: no significant differences between shear viscosity measured at increasing and decreasing shear rate were observed for TUC, while on the other hand, TU solutions show a certain history dependence of viscosity. This was observed at several concentrations, but for clarity only data at the nectar-like level are presented in Fig. 3. Once the fluid has been sheared up to 100 s^−1^, the viscosities measured at decreasing shear rates are significantly lower and the impact on the performance of such fluids during swallowing needs to be considered.

When the viscosities of liquid contrast materials (Fig. 2) and commercial thickeners at different consistencies (Fig. 3) were compared at a shear rate of 50 s^−1^, a close correspondence between Varibar^®^ nectar, E-Z-Paque^®^, TU, and TUC at the nectar-like level was observed. For the other two consistency levels, no direct match was found and even significant differences were registered (e.g., Varibar^®^ honey is around four times more viscous than TUC at the honey-like level at a shear rate of 50 s^−1^). Therefore, the link between diagnosis based on videofluoroscopic examination and prescription of thickened products that are safe to swallow is not always straightforward. One possibility to overcome this constraint could be to use the least viscous contrast material and during videofluoroscopic examination thicken it to desired consistencies using commercial thickeners, which is discussed in the following subsection.

### Matching Viscosities of Diagnostic Fluids and Thickener Solutions

#### An “Easy” Case : Varibar^®^ Thin Liquid and Resource^®^ ThickenUp^TM^ Clear (TUC)

A straightforward application of low-viscosity contrast agent (in this case Varibar^®^ Thin Liquid) thickened to nectar-like level is shown in Fig. 4. The amount of water needed to reconstitute a bottle of Varibar^®^ Thin Liquid was determined beforehand; the corresponding amount of TUC to be added in order to reach the nectar-like consistency was then calculated considering recommended dosages (i.e., 1.2 g TUC powder for 100 ml liquid, see Table 2). The two powders (contrast agent and TUC) were mixed and the product was subsequently reconstituted following supplier instructions. As shown in Fig. 4, the contrast agent thus obtained matched perfectly the viscosity of a thickener solution at nectar-like consistency. The same protocol can be used for preparing contrast agent solutions at all desired consistencies and matching the viscosities of thickened liquids using TUC. Moreover, the viscosity of the thickened contrast agent measured at different times after solution preparation was stable over time (see Fig. 4), allowing great flexibility for the use of this thickened contrast material.

#### A More Difficult Case : Gastrografin^®^ and Resource^®^ ThickenUp^TM^ Clear (TUC)

Depending on the nature of the contrast agent and the thickener used, more complex behavior may occur. As shown in Fig. 2, the viscosity of a solution of Gastrografin^®^ diluted in water at 1:1 volume ratio (recommended by the manufacturer for oral examination in adults) is close to that of water. Nevertheless, when TUC is added to such a solution, the rheological behavior is very different compared to a solution made with pure water as solvent. The viscosities of the TUC/Gastrografin^®^ solutions vary significantly over time (see Figs. 5, 6). The effect is particularly important at shorter times. For example, for a fresh solution (5 min after preparation) the increase in viscosity is so rapid that a controlled measurement of viscosity could not be done (low curve on Fig. 5). During the approximately 4 min of measurement, a clear difference was observed between the upward part of the curve (recorded while increasing the shear rate, right arrow) and the downward part of the curve (recorded while decreasing the shear rate, left arrow). Therefore, the uncertainty of measurement was significant during this build-up phase. For longer times (4- and 12-h data presented in Fig. 5), the viscosity increase was less significant and the two parts of the curve (upward and downward) were identical.

Figure 6 helps to better visualize the buildup of viscosity in solutions of TUC/Gastrografin^®^/water over time and at different concentrations. During the first 3 h after solution preparation, an up to fourfold increase in viscosity can be observed. Thus, for example, a solution that initially corresponded to the thin-liquid range can thicken and reach nectar level viscosities. For longer times (>3 h), the viscosity reaches a plateau and the solutions can then be used with confidence regarding viscosity stability. Nevertheless, the matching of viscosities between thickened videofluoroscopic fluids (i.e., solutions of TUC in Gastrografin^®^/water) and thickener solutions (i.e., TUC in water) is not straightforward. As shown on Fig. 6, depending on TUC concentration, the effect on solution viscosity in the presence of Gastrografin^®^ can vary significantly: at TUC concentrations covering all recommended doses (from nectar to pudding consistencies), solutions prepared with Gastrografin^®^ are significantly *less* viscous than those prepared with pure water. The difference becomes less important as the TUC concentration increases, and for 4.8 g/100 ml this effect is no longer observed. At this specific concentration, the viscosities at 50 s^−1^ of the TUC/water solution and of the TUC/Gastrografin^®^/water solution match perfectly. However, for higher TUC concentrations, the effect on viscosity is reversed: solutions prepared with Gastrografin^®^ become significantly *more* viscous than those prepared with pure water. The nature of the contrast material (iodine compounds) seems to bring complexity in these mixed systems. Further analysis would be needed to better understand and predict this phenomenon, but the iodine-binding capacity of thickeners (like xanthan gum) [24] could be an explanation. These differing effects of concentration and time have to be considered if such solutions are used for videofluoroscopic investigation and subsequent diet prescription.

## Conclusions

The aim of this study was to highlight the importance of accurately characterizing the rheological properties of materials used in the management of persons suffering from dysphagia, be it for diagnosis or diet prescription. When talking about viscosity, one should always keep in mind that apart from truly thin liquids with a viscosity close to that of water, the viscosity depends on the speed of flow or shear rate. Nearly all materials used in this study showed a pronounced decrease of viscosity with shear rate (“shear thinning”). Moreover, the degree of shear thinning can be very different from one material to another, so even if viscosity values are equal at one specific shear rate, they can significantly differ at other shear rates. Such a divergence can lead to different subjective impressions of viscosity for nominally equivalent materials, and in some cases it may affect swallowing itself.

Attention is drawn to the importance of objectively measuring viscosities of diagnostic videofluoroscopic fluids and matching them with liquids prepared with commercially available thickening agents designed specifically for dysphagia patients. Such a “match” is not always straightforward given the variety of contrast agents available on the market, the scarcity of published viscosity data, and the different standards for classifying bolus consistencies. This situation can be improved by systematic use of mixing protocols developed based on reproducible viscosity measurements, such as presented here. Ultimately, for optimal patient outcomes, only diagnostic materials and thickening agents for which reliable viscosity data are available should be used.

## Footnotes

1 A certain (loose) analogy to consider here could be fuel consumption in cars. The liters (or gallons) consumed per kilometer (or mile) depend on the driving speed, and comparing different levels of fuel consumption is meaningful only when referring to the same driving conditions. Likewise, viscosities (force needed to maintain a flow) can only be sensibly compared for equivalent shear rates.

2 It would have been preferable to have the same measurement condition as described in the NDD standard (25 °C), but nevertheless the conclusions of the present study are valid, as not more than 10 % variation in viscosity is measured in this temperature range.
